# A 2100‐km jaguar journey redefines mobility and large‐scale conservation priorities during large carnivore dispersal

**DOI:** 10.1002/ecy.70441

**Published:** 2026-06-16

**Authors:** Jon Morant, Esther Sebastián‐González, Leandro Reverberi Tambosi, Alan Eduardo de Barros, Renato A. Moreira, Francesca Belem Lopes Palmeira, Douglas dos Santos Silva, Tiago J. Silveira, Anah T. A. Jácomo, Leandro Silveira

**Affiliations:** ^1^ Department of Zoology and Animal Cellular Biology University of the Basque Country Araba Spain; ^2^ School of Biology and Environmental Sciences University College Dublin Dublin Ireland; ^3^ University of Alicante Alicante Spain; ^4^ Instituto Multidisciplinar para el Estudio del Medio Ramon Margalef – University of Alicante Alicante Spain; ^5^ Departamento de Ecologia, Instituto de Biociências Universidade de São Paulo São Paulo Brazil; ^6^ Oreades Núcleo de Geoprocessamento Mineiros Brazil; ^7^ RLadies Ribeirão Preto Ribeirão Preto Brazil; ^8^ Instituto Onças do Rio Negro Aquidauana Brazil; ^9^ Jaguar Conservation Fund Mineiros Brazil

**Keywords:** Amazon, endangered species, GPS, jaguar, large predator, movement ecology

On November 27, 2025, we recorded the last GPS location from a 92‐kg adult male jaguar (*Panthera onca*), designated “Gaspar” (Figure 1), marking a pivotal moment that reshapes our understanding of jaguar movement ecology. Gaspar was fitted with a GPS collar (Telonics TGW‐4577‐4 and Iridium Terrestrial System) on November 11, 2024. The GPS collar was set to record locations at 60‐min intervals as established protocols for large carnivore telemetry studies. Gaspar was established in a 697 km^2^ home range along the Araguaia River before initiating an unprecedented 2122‐km journey toward the southwestern region, spanning the border of the Amazon to the inland Cerrado (Figure 2A,B). The 2122 km reported refers to the linear displacement (the straight‐line distance between the start and endpoint of the dispersal phase).

**FIGURE 1:**
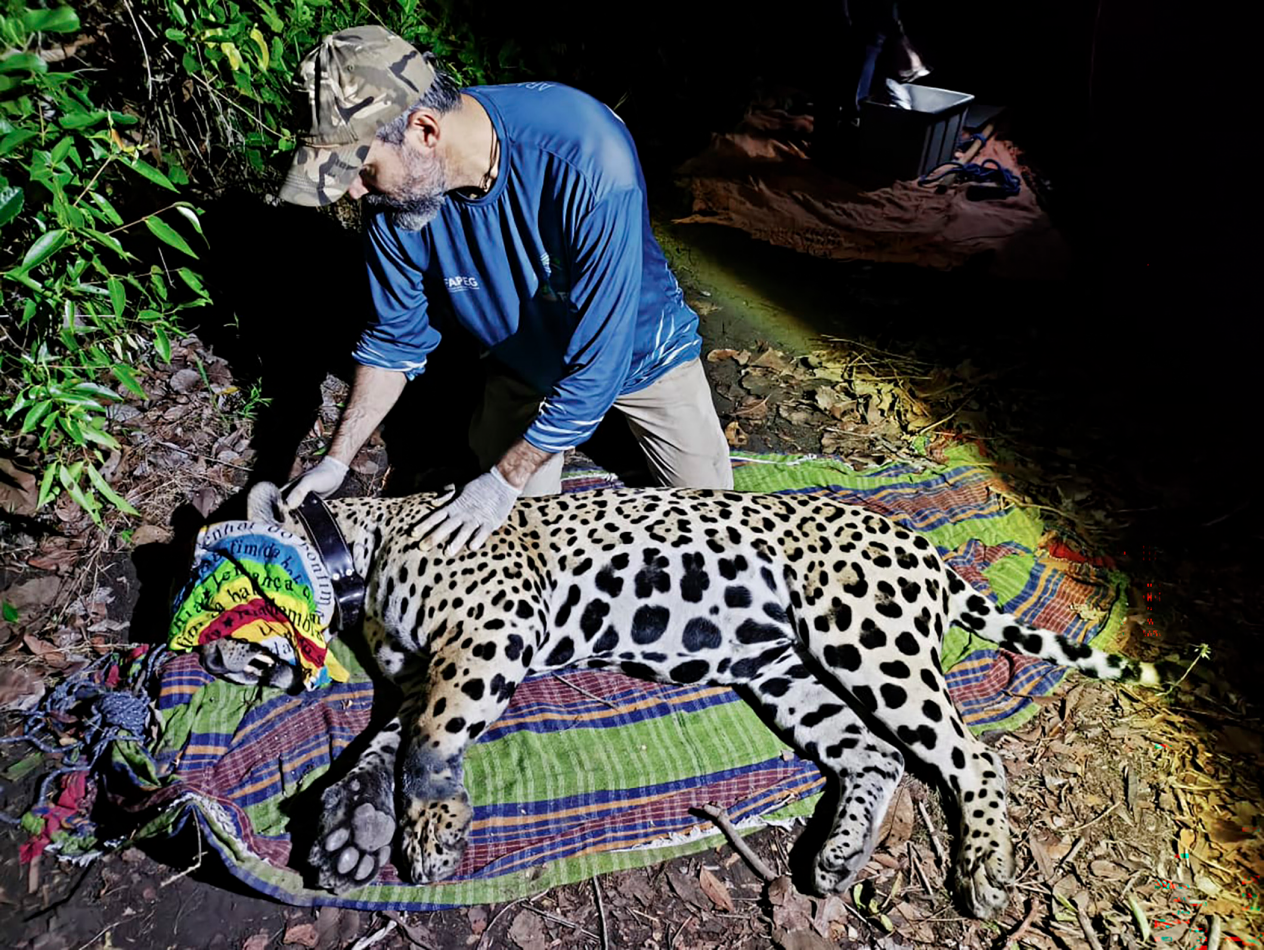
Photograph of “Gaspar,” the male jaguar, during the GPS‐collaring process by Douglas dos Santos Silva (coauthor of the present manuscript). Photo credit: Leandro Silveira.

**FIGURE 2:**
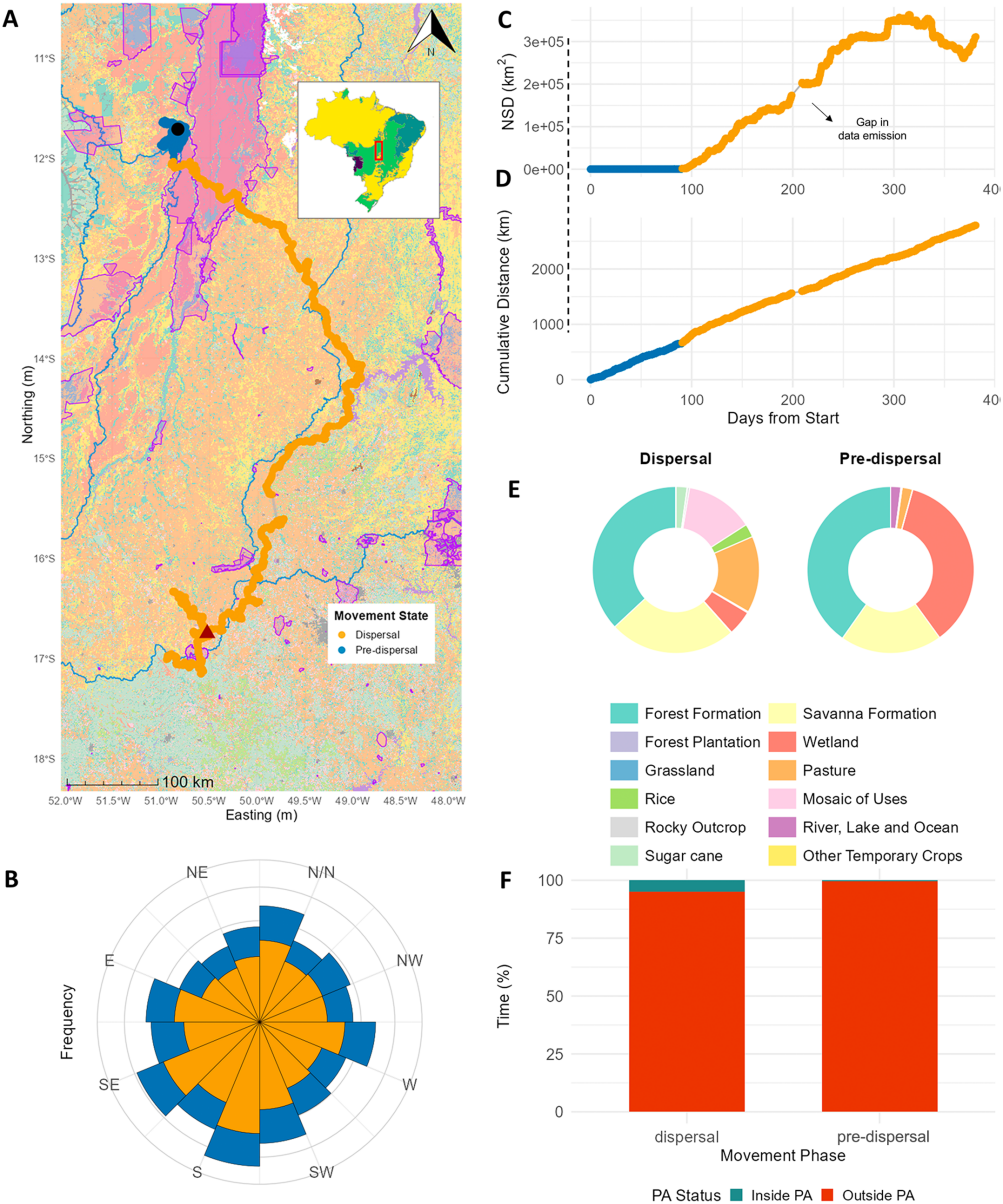
Movement trajectory, behavioral phases, and habitat use of “Gaspar” across approximately 2100 km of tracked linear displacement. (A) Spatial route overlaid on main land cover classes, with movement states classified as dispersal (orange) and pre‐dispersal (blue). Protected areas (PAs) are outlined in purple. The inset locates the study area within Brazil, with major biomes color‐coded: Amazon (yellow), Cerrado (green), Atlantic Forest (blue‐green), Caatinga (blue), Pantanal (purple), and Pampa (light yellow). (B) Rose diagram of movement direction for the dispersal and pre‐dispersal phases (same color scheme as in panel A). (C) Net squared displacement (NSD) and (D) cumulative distance (km) through time, both relative to the start of monitoring; the dashed line marks the onset of dispersal, and the arrow indicates a gap in data transmission. (E) Proportional time use of land cover classes during dispersal and pre‐dispersal. (F) Proportion of time spent inside (blue) versus outside (red) PAs during each phase.

Gaspar was part of our ongoing project based in Brazil, which utilizes high‐frequency GPS telemetry to monitor the spatial behavior of jaguars along the Araguaia River, a potential large‐scale corridor between the Cerrado grasslands and the Amazonian forest, within a broader Brazil‐wide framework of corridor planning for jaguar conservation (Silveira et al., 2014). His data are contributing to the expanding global database of carnivore movement patterns (Jędrzejewski et al., 2018; Morato et al., 2018). Gaspar's movement was recorded over 5050 GPS locations. We classified movement phases following established criteria (Edelhoff et al., 2016): the pre‐dispersal phase (~27% of locations) comprised the period of residency within the established home range along the Araguaia River, characterized by area‐restricted, tortuous movement; the dispersal phase (~73% of locations) began with a sustained directional shift away from the home range and continued until the last recorded location. Throughout the 381‐day dispersal phase alone, Gaspar traveled at an average of 9.76 km/day and a mean speed of 0.407 km/h, moving mainly toward the south–southwest (Figure 2C,D). Although these daily movement rates are somewhat lower than the maximum values reported for some jaguars and other large carnivores in Neotropical systems (e.g., 14–18 km/day for “Xangô” in the Cerrado and >15 km/day for two individuals in the Atlantic Forest; Morato et al., 2016; up to 28.8 km/day for an individual in the Dry Chaco; McBride & Thompson, 2018), Gaspar's trajectory is notable for the unusually long and sustained dispersal episode, with consistent long‐distance movements maintained over several months rather than brief bursts of high daily displacement.

Comparative analysis with other large carnivore systems provides additional context for Gaspar's exceptional movement. Recent documentation of transfrontier carnivore dispersals in southern Africa recorded movements of 550–800 km for African wild dogs and lions (Goodheart et al., 2022), while European brown bears and wolves regularly disperse 200–500 km (Swenson et al., 2017). However, Gaspar's 2100 km displacement represents one of the longest recorded dispersal events for any Neotropical felid. This substantially exceeds documented jaguar dispersal distances of >50 km (Morato et al., 2016) and is comparable to the longest cougar dispersal records, including a male that traveled >2700 km from South Dakota to Connecticut (Hawley et al., 2016) and a female that dispersed 1341 km (Stoner et al., 2008).

This sustained, directional, and consistent movement surpasses the previous record of 315 km documented in a subadult tiger (*Panthera tigris*) in India (Hussain et al., 2022), and far exceeds typical monthly jaguar distances (~120 km) reported elsewhere (Foster et al., 2010; Hutchinson et al., 2023). Recent studies of jaguar space use across five biomes in Brazil and Argentina have documented maximum home ranges of approximately 150–1200 km^2^, depending on the region (McBride & Thompson, 2018; Morato et al., 2016), making Gaspar's 2122‐km linear displacement truly exceptional. Such large‐scale mobility situates jaguars within the realm of highly vagile terrestrial mammals, comparably mobile to species such as caribou and gray wolves (Joly et al., 2019), and approaching the movement scales of African carnivores, including lions and wild dogs, that regularly traverse 200–800 km during dispersal (Beytell et al., 2024; Sandoval‐Serés et al., 2022).

This individual spent comparatively more time in forested areas and Savannas during the pre‐dispersal phase; however, during the dispersal phase, he made proportionally greater use of human‐modified habitats, such as pastures and plantations (e.g., rice and sugarcane), while still spending the majority of time in natural vegetation (Figure 2E). His movement highlights remarkable persistence and behavioral plasticity in the face of anthropogenic barriers and habitat fragmentation. During the dispersal phase, Gaspar modified his habitat selection to navigate a heavily modified landscape, increasing his use of agricultural matrices while maintaining directional progress toward the southwest, which is a pattern consistent with opportunistic route selection rather than targeted navigation toward a specific destination (Figure 2F). Contemporary movement ecology research emphasizes the importance of environmental heterogeneity in shaping carnivore dispersal patterns (Tucker et al., 2018), with recent GPS‐based studies revealing how large felids adapt their behavior across different land‐use contexts (Alegre et al., 2024). Recent analyses of jaguar GPS data demonstrate behavioral plasticity during movement, with animals exhibiting different habitat preferences during local versus long‐distance movements (Schoen et al., 2025).

Although the Amazon and Pantanal remain critical reservoirs for jaguar populations, they are increasingly threatened by deforestation, agricultural expansion, and infrastructure development, mirroring pressures that have long been observed in the Cerrado and Atlantic Forest (Barlow et al., 2016; Ferreira et al., 2014). Recent evidence emphasizes the fragility of Brazil's forest ecosystems and the cascading impacts on carnivore health and persistence (Bogoni et al., 2023). The Amazon alone has lost over 33% of its original natural areas, including forests (Mapbiomas, 2025). In the Cerrado, agricultural conversion has eliminated 46% of native vegetation, resulting in a highly fragmented landscape that impedes the movement of large carnivores (Strassburg et al., 2017). These trends are especially relevant, as the Amazon and Pantanal are strongholds for the species and encompass the core jaguar populations, while the Cerrado and, even more so, the Atlantic Forest maintain smaller and more isolated populations. Hence, connectivity maintenance across these landscapes is essential for gene flow and demographic stability at species‐range scales, a need that is amplified in increasingly human‐dominated environments (Crooks et al., 2017).

From a conservation perspective, this event challenges current spatial management paradigms that rely on limited home range data and often neglect long‐distance movements (Allen & Singh, 2016). Traditional protected area (PA) designs may be insufficient for species exhibiting such extensive movement patterns (Newmark, 2008; Figure 2F). Moreover, PAs alone are insufficient to ensure the persistence of large carnivores in anthropogenic landscapes (Torres‐Romero et al., 2025). Our findings suggest that, while structural corridors are vital, maintaining “landscape permeability” in the anthropogenic matrix is equally critical and more intensively used during dispersal, including pastures and croplands such as rice and sugarcanes (Figure 3). We define landscape permeability here as the degree to which human‐modified land uses allow safe passage for dispersing individuals without lethal persecution or insurmountable barriers. For movements of this magnitude, jaguars rely on a mosaic of working landscapes (e.g., cattle ranches with forest remnants) rather than continuous pristine corridors (Zeller et al., 2012). This further emphasized species plasticity in resource acquisition while navigating anthropogenic landscapes (e.g., livestock, small mammals, opportunistic feeding). Thus, conservation efforts must focus on reducing mortality risk (e.g., through co‐existence programs and livestock protection measures) in these human‐dominated areas to ensure functional connectivity, including measures to reduce retaliatory killing (Marchini & Macdonald, 2012), hunting (Jędrzejewski et al., 2017), and poisoning (Csermak et al., 2023) and even main prey depletion due to overhunting (Romero‐Muñoz et al., 2019).

**FIGURE 3:**
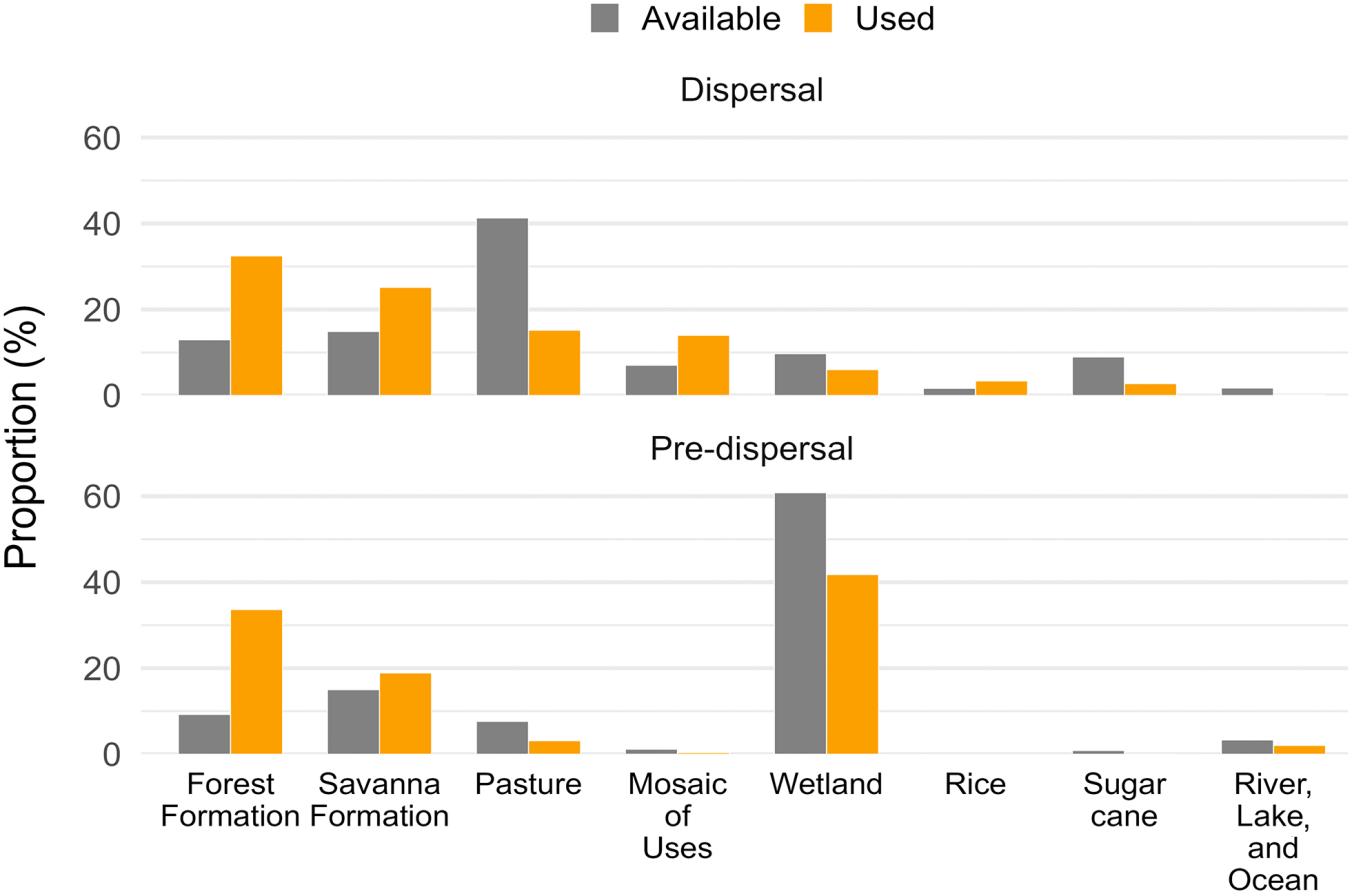
Proportion of available and used land cover types during each movement phase of “Gaspar.” GPS fixes were classified by behavioral phase (pre‐dispersal, dispersal) and the proportion falling within each land cover class to quantify habitat use. Habitat availability was estimated as the proportional pixel composition of each land cover class within a buffer of 2000 m around the phase‐specific trajectory. Land cover classes and biome boundaries are from MapBiomas Collection 9 (MapBiomas Project, 2025).

Recent legislative developments in Brazil provide some optimism for maintaining connectivity pathways essential for movements like Gaspar's. In 2024, ecological corridors in the Pantanal gained enhanced recognition and planning guidance under Mato Grosso do Sul Decree No. 16.388, which complements Law No. 6.160 (2023). These legal instruments define ecological corridors and emphasize their ecological importance, offering guidelines that align corridor management with the Brazilian Forest Code (Law No. 12.651). However, their implementation remains challenging, as human activities overlap with 29%–70% of identified carnivore movement corridors in the region (Montalvo et al., 2023). Notably, these regulatory advances are limited to the Pantanal, highlighting the need for similar legislative frameworks across Brazil and throughout the jaguar's broader range to ensure effective ecological connectivity.

The timing of Gaspar's journey may be influenced by ecological drivers, such as resource distribution, mating opportunities, or anthropogenic disturbance. However, the age of Gaspar at dispersal onset (~8 years) challenges the typical “natal dispersal” hypothesis observed in subadults. Instead, this movement likely represents a case of “breeding dispersal” or secondary displacement (Greenwood, 1980), possibly triggered by the loss of territory to a competitor or local density‐dependent factors. This observation prompts new questions about large carnivore mobility, particularly in adults, suggesting that mature individuals may function as critical long‐distance genetic linkers between isolated populations (Harmsen et al., 2017).

Our findings provide a compelling case for large‐scale, integrative conservation approaches that accommodate the dynamic and extensive spatial requirements of highly endangered apex carnivores, such as jaguars (De Angelo et al., 2011). Although based on a single individual, the documentation of this extraordinary dispersal event raises important questions about the spatial scales at which jaguar conservation must operate and underscores the potential value of transnational conservation frameworks that can maintain landscape permeability across the species’ range (Paviolo et al., 2016; Villalva & Palomares, 2022). As habitat fragmentation accelerates across the Neotropics, understanding and protecting the movement corridors revealed by individuals like “Gaspar” becomes increasingly critical for jaguar population persistence and the maintenance of ecosystem integrity across South America's most biodiverse landscapes.

## FUNDING INFORMATION

This research was supported by the Jaguar Conservation Fund as part of the jaguar corridor project in Brazil. JM was supported by the Basque Government Postdoctoral grant (POS_2025_1_0009). LRT was supported by a Maria Zambrano grant co‐funded by the Ministry of Universities of Spain, by the European Union—Next Generation program, and by University of Alicante and by a FAPESP‐NSF research project (2022/02174‐5), by the Coordenação de Aperfeiçoamento de Pessoal de Nível Superior—Brasil (CAPES)—Finance Code 001 and Fundação de Amparo à Pesquisa do Estado de São Paulo—FAPESP process codes 2018/24891‐5 and 2022/02174‐5. ESG received grants RYC2019‐027216‐I and CNS2023‐144791 funded by the Spanish Ministry of Science, Innovation and Universities (MCIN/AEI/https://doi.org/10.13039/501100011033) and by ESF Investing in Your Future.

## CONFLICT OF INTEREST STATEMENT

The authors declare no conflicts of interest.

## Data Availability

De‐identified GPS telemetry data (Morant Etxebarria, 2026) are available in Figshare at https://doi.org/10.6084/m9.figshare.32300463.v1. Considering the sensitivity around jaguar locations, exact GPS coordinates and movement analysis code can only be made available to qualified researchers by contacting the data owner (Leandro Silveira; email: l.silveira@jaguar.org.br).

## References

[ecy70441-bib-0033] Alegre, V. B. , C. Z. Kanda , J. E. de Faria Oshima , B. B. Niebuhr , R. G. Morato , J. J. Thompson , L. Börger , et al. 2024. “Jaguar at the Edge: Movement Patterns in Human‐Altered Landscapes.” Perspectives in Ecology and Conservation 22, no. 4: 358–366.

[ecy70441-bib-0001] Allen, A. M. , and N. J. Singh . 2016. “Linking Movement Ecology with Wildlife Management and Conservation.” Frontiers in Ecology and Evolution 3: 155.

[ecy70441-bib-0002] Barlow, J. , G. D. Lennox , J. Ferreira , E. Berenguer , A. C. Lees , R. M. Nally , J. R. Thomson , et al. 2016. “Anthropogenic Disturbance in Tropical Forests Can Double Biodiversity Loss from Deforestation.” Nature 535: 144–147. 27362236 10.1038/nature18326

[ecy70441-bib-0039] Beytell, P. , L. Hanssen , O. Aschenborn , and R. Naidoo . 2024. “Long‐Distance, Transfrontier Carnivore Dispersals in Southern Africa.” Ecology and Evolution 14(11): e70574. 39530035 10.1002/ece3.70574 PMC11554372

[ecy70441-bib-0003] Bogoni, J. A. , V. Boron , C. A. Peres , M. E. M. Coelho , R. G. Morato , and M. Oliveira‐da‐Costa . 2023. “Impending Anthropogenic Threats and Protected Area Prioritization for Jaguars in the Brazilian Amazon.” Communications Biology 6: 132. 36792802 10.1038/s42003-023-04490-1 PMC9932174

[ecy70441-bib-0004] Crooks, K. R. , C. L. Burdett , D. M. Theobald , S. R. B. King , M. di Marco , C. Rondinini , and L. Boitani . 2017. “Quantification of Habitat Fragmentation Reveals Extinction Risk in Terrestrial Mammals.” Proceedings of the National Academy of Sciences 114: 7635–7640. 10.1073/pnas.1705769114 PMC5530695 28673992

[ecy70441-bib-0005] Csermak, A. C. , G. R. De Araújo , C. S. Pizzutto , T. de Deco‐Souza , and P. N. Jorge‐Neto . 2023. “GPS Collars as a Tool to Uncover Environmental Crimes in Brazil: The Jaguar as a Sentinel.” Animal Conservation 26(2): 137–139.

[ecy70441-bib-0006] De Angelo, C. , A. Paviolo , and M. Di Bitetti . 2011. “Differential Impact of Landscape Transformation on Pumas and Jaguars in the Upper Paraná Atlantic Forest.” Diversity and Distributions 17: 422–436.

[ecy70441-bib-0037] Edelhoff, H. , J. Signer , and N. Balkenhol . 2016. “Path Segmentation for Beginners: An Overview of Current Methods for Detecting changes in Animal Movement Patterns.” Movement Ecology 4(1): 21. 27595001 10.1186/s40462-016-0086-5 PMC5010771

[ecy70441-bib-0009] Ferreira, J. , L. E. Aragão , J. Barlow , P. Barreto , E. Berenguer , M. Bustamante , T. A. Gardner , et al. 2014. “Brazil's Environmental Leadership at Risk.” Science 346, no. 6210: 706–707. 25378611 10.1126/science.1260194

[ecy70441-bib-0041] Foster, R. J. , B. J. Harmsen , and C. P. Doncaster . 2010. “Habitat Use by Sympatric Jaguars and Pumas Across a Gradient of Human Disturbance in Belize.” Biotropica 42(6): 724–731.

[ecy70441-bib-0010] Goodheart, B. , S. Creel , M. A. Vinks , K. Banda , J. Reyes de Merkle , A. Kusler , C. Dart , et al. 2022. “African Wild Dog Movements Show Contrasting Responses to Long and Short Term Risk of Encountering Lions: Analysis Using Dynamic Brownian Bridge Movement Models.” Movement Ecology 10: 16. 35361272 10.1186/s40462-022-00316-7 PMC8974231

[ecy70441-bib-0011] Greenwood, P. J. 1980. “Mating Systems, Philopatry and Dispersal in Birds and Mammals.” Animal Behaviour 28: 1140–1162.

[ecy70441-bib-0012] Harmsen, B. J. , R. J. Foster , S. C. Silver , L. E. T. Ostro , and C. P. Doncaster . 2017. “Jaguar and Puma Activity Patterns in Relation to their Main Prey.” Mammalian Biology 84: 1–11.

[ecy70441-bib-0013] Hawley, J. E. , P. W. Rego , A. P. Wydeven , M. K. Schwartz , T. C. Viner , R. Kays , K. L. Pilgrim , and J. A. Jenks . 2016. “Long‐Distance Dispersal of a Subadult Male Cougar from South Dakota to Connecticut Documented with DNA Evidence.” Journal of Mammalogy 97: 1435–1440.

[ecy70441-bib-0038] Hussain, Z. , P. Ghaskadbi , P. Panchbhai , R. Govekar , P. Nigam , and B. Habib . 2022. “Long‐Distance Dispersal by a Male Sub‐Adult Tiger in a Human‐Dominated Landscape.” Ecology and Evolution 12(9): e9307. 36188506 10.1002/ece3.9307 PMC9514059

[ecy70441-bib-0040] Hutchinson, E. , H. Hausermann , and Z. Walder‐Hoge . 2023. “A Spatial Analysis of Border “Security” and Jaguars in the US–Mexico Borderlands.” Frontiers in Conservation Science 4: 1355997.

[ecy70441-bib-0015] Jędrzejewski, W. , H. S. Robinson , M. Abarca , K. A. Zeller , G. Velasquez , E. A. D. Paemelaere , J. F. Goldberg , et al. 2018. “Estimating Large Carnivore Populations at Global Scale Based on Spatial Predictions of Density and Distribution—Application to the Jaguar (*Panthera onca*).” PLoS One 13: e0194719. 29579129 10.1371/journal.pone.0194719 PMC5868828

[ecy70441-bib-0014] Jędrzejewski, W. , R. Carreño , A. Sánchez‐Mercado , K. Schmidt , M. Abarca , H. S. Robinson , E. O. Boede , et al. 2017. “Human‐Jaguar Conflicts and the Relative Importance of Retaliatory Killing and Hunting for Jaguar (*Panthera onca*) Populations in Venezuela.” Biological Conservation 209: 524–532.

[ecy70441-bib-0016] Joly, K. , L. Börger , and S. Henley . 2019. “Longest Terrestrial Migrations and Movements Around the World.” Scientific Reports 9: 14662. 31654045 10.1038/s41598-019-51884-5 PMC6814704

[ecy70441-bib-0017] MapBiomas . 2025. “Project MapBiomas—Collection 10 of the Annual Land Cover and Land Use Maps of Brazil (1985–2024).” https://brasil.mapbiomas.org/

[ecy70441-bib-0018] Marchini, S. , and D. W. Macdonald . 2012. “Predicting Ranchers' Intention to Kill Jaguars: Case Studies in Amazonia and Pantanal.” Biological Conservation 147: 213–221. 10.1016/j.biocon.2012.01.002.

[ecy70441-bib-0019] McBride, R. T., Jr. , and J. J. Thompson . 2018. “Space Use and Movement of Jaguar (*Panthera onca*) in Western Paraguay.” Mammalia 82: 97–104.

[ecy70441-bib-0021] Montalvo, V. H. , C. Sáenz‐Bolaños , E. Carrillo , and T. K. Fuller . 2023. “A Review of Environmental and Anthropogenic Variables Used to Model Jaguar Occurrence.” Neotropical Biology and Conservation 18: 31–51.

[ecy70441-bib-0022] Morant Etxebarria, J. 2026. “Data For: A 2100‐Km Jaguar Journey Redefines Mobility and Large‐Scale Conservation Priorities During Large Carnivore Dispersal.” Figshare. Dataset. 10.6084/m9.figshare.32300463.v1 PMC13270777 42300677

[ecy70441-bib-0023] Morato, R. G. , J. A. Stabach , C. H. Fleming , J. M. Calabrese , R. C. de Paula , K. M. P. M. Ferraz , D. L. Z. Kantek , et al. 2016. “Space Use and Movement of a Neotropical Top Predator: The Endangered Jaguar.” PLoS One 11: e0168176. 28030568 10.1371/journal.pone.0168176 PMC5193337

[ecy70441-bib-0024] Morato, R. G. , J. J. Thompson , A. Paviolo , J. A. de la Torre , F. Lima , R. T. McBride, Jr. , R. C. Paula , et al. 2018. “Jaguar Movement Database: A GPS‐Based Movement Dataset of an Apex Predator in the Neotropics.” Ecology 99: 1691. 29961270 10.1002/ecy.2379

[ecy70441-bib-0025] Newmark, W. D. 2008. “Isolation of African Protected Areas.” Frontiers in Ecology and the Environment 6: 321–328.

[ecy70441-bib-0026] Paviolo, A. , C. de Angelo , K. M. P. M. B. Ferraz , R. G. Morato , J. Martinez Pardo , A. C. Srbek‐Araujo , B. M. Beisiegel , et al. 2016. “A Biodiversity Hotspot Losing its Top Predator: The Challenge of Jaguar Conservation in the Atlantic Forest of South America.” Scientific Reports 6: 37147. 27849006 10.1038/srep37147 PMC5111070

[ecy70441-bib-0027] Romero‐Muñoz, A. , R. Torres , A. J. Noss , A. J. Giordano , V. Quiroga , J. J. Thompson , M. Baumann , et al. 2019. “Habitat Loss and Overhunting Synergistically Drive the Extirpation of Jaguars from the Gran Chaco.” Diversity and Distributions 25(2): 176–190.

[ecy70441-bib-0042] Sandoval‐Serés, M. E. , W. Moyo , D. Madhlamoto , H. Madzikanda , P. Blinston , R. Kotze , E. van der Meer , and A. Loveridge . 2022. “Long‐Distance African Wild Dog Dispersal Within the Kavango‐Zambezi Transfrontier Conservation Area.” African Journal of Ecology 60(4): 13065.

[ecy70441-bib-0028] Schoen, J. M. , R. DeFries , and S. Cushman . 2025. “Open‐Source, Environmentally Dynamic Machine Learning Models Demonstrate Behavior‐Dependent Utilization of Mixed‐Use Landscapes by Jaguars (*Panthera onca*).” Biological Conservation 301: 110585.

[ecy70441-bib-0029] Silveira, L. , R. Sollmann , A. T. A. Jácomo , J. A. F. Diniz Filho , and N. M. Tôrres . 2014. “The Potential for Large‐Scale Wildlife Corridors between Protected Areas in Brazil Using the Jaguar as a Model Species.” Landscape Ecology 29(7): 1213–1223.

[ecy70441-bib-0030] Stoner, D. C. , W. R. Rieth , M. L. Wolfe , M. B. Mecham , and A. N. N. Neville . 2008. “Long‐Distance Dispersal of a Female Cougar in a Basin and Range Landscape.” Journal of Wildlife Management 72: 933–939.

[ecy70441-bib-0031] Strassburg, B. B. N. , T. Brooks , R. Feltran‐Barbieri , A. Iribarrem , R. Crouzeilles , R. Loyola , A. E. Latawiec , et al. 2017. “Moment of Truth for the Cerrado Hotspot.” Nature Ecology & Evolution 1: 0099. 10.1038/s41559-017-0099 28812670

[ecy70441-bib-0032] Swenson, J. E. , M. Schneider , A. Zedrosser , A. Söderberg , R. Franzén , and J. Kindberg . 2017. “Challenges of Managing a European Brown Bear Population; Lessons from 30 Years of Research.” Wildlife Biology 2017: wlb.00251.

[ecy70441-bib-0034] Torres‐Romero, E. J. , T. M. Eppley , W. J. Ripple , T. M. Newsome , M. Krofel , N. H. Carter , A. Ordiz , T. G. de Oliveira , N. Selva , and V. Penteriani . 2025. “Global Scale Assessment of the Human‐Induced Extinction Crisis of Terrestrial Carnivores.” Science Advances 11: eadq2853. 40668902 10.1126/sciadv.adq2853 PMC12266096

[ecy70441-bib-0035] Tucker, M. A. , K. Böhning‐Gaese , W. F. Fagan , J. M. Fryxell , B. van Moorter , S. C. Alberts , A. H. Ali , et al. 2018. “Moving in the Anthropocene: Global Reductions in Terrestrial Mammalian Movements.” Science 359: 466–469. 29371471 10.1126/science.aam9712

[ecy70441-bib-0020] Villalva, P. , and F. Palomares . 2022. “A Continental Approach to Jaguar Extirpation: A Tradeoff Between Anthropic and Intrinsic Causes.” Journal for Nature Conservation 66: 126145.

[ecy70441-bib-0036] Zeller, K. A. , K. McGarigal , and A. R. Whiteley . 2012. “Estimating Landscape Resistance to Movement: A Review.” Landscape Ecology 27: 777–797.

